# Green Synthesis of CuO Nanoparticles Using *Beta vulgaris* L. Extract for Removal of Methyl Violet Dye

**DOI:** 10.1155/tswj/4954329

**Published:** 2026-05-20

**Authors:** Muna Abd Ul Rasool AL-Kazragi, Dhafir T. A. AL-Heetimi, Amal Khudair Al-Jaafari, Lee D. Wilson

**Affiliations:** ^1^ Department of Chemistry, College of Education for Pure Science Ibn-Al-Haitham, University of Baghdad, Baghdad, Iraq, uobaghdad.edu.iq; ^2^ Al-Rusafa Second Directorate of Education-Iraqi Ministry of Education, Baghdad, Iraq; ^3^ Department of Chemistry, University of Saskatchewan, Saskatoon, Canada, usask.ca

**Keywords:** adsorption mechanism, dye adsorption capacity, equilibrium thermodynamics, methyl violet, nanoparticles

## Abstract

Wastewater discharge containing organic dyes may pose a hazard to the environment, which necessitates that dye removal must occur prior to wastewater release into water bodies. Herein, copper oxide nanoparticles (CuO NPs) were prepared by a green precipitation method to enable decolorization of a cationic dye (methyl violet; MV) from aqueous media. Complementary tools were employed to characterize the CuO NPs adsorbent: spectroscopy (FTIR and UV‐VIS), microscopy (FESEM and TEM), XRD, BET surface area analysis, and point of zero charge (pH_PZC_) via potentiometry. The FTIR bands at 722, 663, 569, and 465 cm^−1^ correspond to the vibrational modes of CuO NPs, along with the optical absorbance band at 275 nm that supports the formation of CuO NPs. The XRD and TEM analyses predicted single‐phase CuO NPs with a monoclinic framework. BET was employed to assess the textural characteristics and accounted for the specific surface area (12.97 m^2^·g^−1^). Batch adsorption studies were carried out to assess the role of initial pH (3.58–10.53), CuO NPs dose (0.02–0.25 g/L), initial MV concentration (20–140 mg/L), contact time (5–90 min), and temperature (298, 308, and 318 K) on the dye removal efficiency. The adsorption capacity of CuO NPs for MV was determined to be 5.06 mg/g at 45°C. The pseudo‐second‐order (PSO) model described kinetic isotherms, and equilibrium adsorption data were adequately fitted by the Freundlich model. Thermodynamic results revealed that adsorption was spontaneous, endothermic, and entropy driven at the solid–liquid interface. The CuO NPs further displayed good reusability with high efficiency for six successive cycles of adsorption–desorption using 0.1 M HCl as a desorbing agent. These findings validate the efficacy of CuO NPs as a green and effective adsorbent for wastewater treatment processes for cationic dye removal.

## 1. Introduction

Nanotechnology has been developing rapidly worldwide, making possible a broad array of applications across various fields, such as materials science to medicine. The unique properties of NPs have been applied in numerous areas, such as biomedical devices, renewable energy, and environmental remediation. Metal oxide nanoparticles (NPs) with their controllable size and composition have drawn attention for addressing challenges in biomedical, technological, and environmental applications. To mitigate the environmental limitations of traditional NP synthesis, new emerging green technologies are under development by applying the principles of cleaner production to reduce environmental damage [1]. Green synthesis of NPs using plant extracts (leaves and fruits) or biological organisms (bacteria, seaweed, yeast, and fungi) presents a viable alternative owing to its low cost, high yield, and eco‐friendly conditions [2]. Overall, the application of green‐synthesized NPs for wastewater treatment offers great potential for sustainable, large‐scale elimination of pollutants.

In 2023, a UNESCO report highlights that nearly 26% of the world′s population lacks access to clean drinking water, revealing scarcity as a serious global concern. The statistics confirm that 2–3 billion individuals currently do not have sufficient clean water. If no measure is taken to address pollution, the situation will worsen over the coming years. The presence of a vast array of pollutants, namely, heavy metal ions, dyes, toxic chemicals, fluoride, phosphates, nitrates, and pharmaceutical residues in water bodies reduces the quality of water [3]. Among these chemicals, dyes are listed as major pollutants in water and wastewater, which originate from industrial effluents such as textile manufacturing, pigment synthesis, leather processing, cosmetics, printing, paper mills, and food processing. Colored industrial effluents, even at low concentrations, can render water unsuitable for use and cause serious environmental issues. Dyes make water appear cloudier, prevent sunlight from penetrating, display ecosystem toxicity, and hinder photosynthetic processes [4]. Furthermore, dyes are stable and can bio‐accumulate in the food chain, causing serious risks to public health. They are associated with genetic mutations, skin allergic responses, gastrointestinal problems, fatigue, cancer, and kidney, liver, brain, reproductive, and nervous system failure. Industrial dyes are usually divided into three groups: cationic (basic dyes), anionic (direct, acid, and reactive dyes), and nonionic (dispersed dyes). Methyl violet (MV), a cationic dye, is widely used in many areas because of its convenience, appropriate adherence to materials, and stability. MV has been applied in medical and veterinary practice as a bacteriostatic agent, disinfectant, and histological stain. This dye is extremely toxic and can induce ailments like carcinogenicity, blood coagulation, gastroenteritis, skin irritation, vision loss, and respiratory complications. MV readily adsorbs onto negatively charged cell membranes, penetrates the cells, and becomes deposited within the cytoplasm. Due to the serious health and environmental hazards created by MV, it is important to eliminate this dye from wastewater prior to its release into the environment [5]. Consequently, removal of dyes from wastewater becomes mandatory, prior to discharge into the environment. Physico‐chemical methods such as ion exchange [6], ozonation [7], Fenton′s oxidation [8], and photocatalytic degradation by irradiation are techniques that have been extensively employed for the decolorization of dyes from synthetic and industrial wastewaters. Biological treatments involving the utilization of microbial fuel cells [9], fungi [10], algae [11], and enzymes have been reported [12]. These treatments result in the formation of secondary pollutants, which create further environmental concern. By comparison, adsorption is a very good remediation technique owing to the ease of handling, affordability, low infrastructure requirements, environmental friendliness, and high efficiency for a broad spectrum of pollutant concentrations [13], etc. Diverse materials with variable adsorption properties have been studied, as follows: coffee waste [14], chitosan [15, 16], clay minerals (natural and modified) [17], and magnetic NPs [18]. Copper oxide nanoparticles (CuO NPs) can be synthesized using various techniques, including solvothermal, chemical vapor deposition, sol–gel, and laser ablation. However, these conventional methods are often limited by the use of hazardous chemicals, high costs, and considerable energy demands. A simple and promising alternative is the green synthesis of CuO NPs using natural precursors. Due to its low energy consumption, cost‐effectiveness, and scalability for high‐yield production, this approach is particularly attractive for water treatment applications [19, 20].

CuO NPs were chosen due to their catalytic activity towards the removal of pollutants based on their unique properties, like low cost and suitability for photocatalytic treatment. Nevertheless, CuO NPs can degrade and release radicals during treatment of wastewater, causing oxidative stress to living organisms. Their environmental impact is influenced by various factors (particle concentration, size, and morphology), where the consequences of their release in large‐scale systems remain unclear. Continued investigation into the ecotoxicological effects of CuO NPs is essential, particularly given the current lack of comprehensive data on human exposure through treated water [21]. CuO NPs as adsorbent for decontamination of dyes have also been the point of limited studies. Indeed, CuO NPs get a lesser diffusion route than traditional adsorbents like activated carbon, resulting in great adsorption capacity and rapid adsorption kinetics. Hence, it was observed that CuO NPs can efficiently eliminate environmental pollutants due to their simplicity of preparation and nontoxicity. Batool et al. prepared CuO NPs using *Camellia sinensis* leaves extract as a reductant and CuSO_4_·5H_2_O as the precursor salt to remove Congo red and Malachite green from aqueous solutions [22]. To synthesize CuO NPs, Davarnejad et al. employed *Centaurea cyanus* plant extract as the reducing agent. In this example, copper sulfate pentahydrate salt was applied as the precursor. The created CuO NPs were used to adsorb methylene blue from water [23]. While Anchani et al. synthesized CuO NPs from banana blossom and E‐waste by chemical process, the precursor salt was copper sulfate in the present study. The synthesized CuO NPs were applied to eliminate MV dye from aqueous solutions [24]. The synthesis of CuO NPs from *Beta vulgaris* L. is not recorded in the literature. The precipitation method was chosen because of its simplicity, economic efficiency, and rapid reaction rate at low temperatures [25].

Beetroot (*Beta vulgaris* L.), a member of the Amaranthaceae family, is widely consumed globally due to its high concentration of bioactive compounds, including polyphenols, inorganic nitrates, and betalains, along with essential minerals and vitamins contained in its fleshy root. People often eat it as a vegetable, and its juice is used as a natural food color, a cosmetic additive, and a part of traditional medicine to stop and treat heart disease, cancer, and diabetes. Beetroot also has strong anti‐inflammatory and antioxidant properties [26].

The main objective of the present study is to evaluate the efficiency of a green strategy for the synthesis of CuO NPs from *Beta vulgaris* L. for the adsorption of MV dye from aqueous solution. The attainment of maximum removal of dye by determining optimum conditions of influential variables (CuO NPs dose, contact time, temperature, and pH) was systematically evaluated. In turn, adsorption kinetics and thermodynamic investigations were also performed to characterize the dye adsorption properties and mechanism of the dye adsorption process. Finally, the aim of this work is to reveal the method and outcomes of the green synthesis of CuO NPs to contribute to the literature of this field and to help researchers in the field.

## 2. Experimental

### 2.1. Materials

The materials used in this study included copper nitrate trihydrate [Cu(NO_3_)_2_·3H_2_O], sodium hydroxide (NaOH), deionized water, and MV (C_24_H_28_N_3_Cl; molecular weight: 393.958 g/mol), all of which were obtained with high purity from Merck, Germany. The *Beta vulgaris* L. was attained from an Iraqi local market.

### 2.2. Green Synthesis of CuO NPs

The collected fresh *Beta vulgaris* L. were submitted to washing with deionized water to remove fine dust particles and cutting into small pieces, then, dropped into 100 mL of deionized water in a beaker at 50°C for 30 min. Consequently, the extract was filtered through Whatman filter paper (42‐mm diameter) to eliminate solid impurities. The clear aqueous extract was kept in a closed glass flask in a refrigerator for future use.

CuO NPs were prepared by a precipitation approach applying a green method. In 20 mL of deionized water, 0.01 mol of copper nitrate was dissolved and magnetically stirred at room temperature for 30 min. Then, 40 mL of *Beta vulgaris* L. extract was added to the solution, resulting in a color change from blue to a deep red with constant stirring. Then, in the above solution, 50 mL of 0.5 M NaOH was added slowly while continually stirring until a dark brown precipitate is attained. The dark brown color precipitate denotes the creation of CuO NPs, which were left to settle for 24 h. Finally, the resulted CuO NPs are washed with deionized water several times to remove any impurities. Afterwards, the precipitate was dried in an oven at 120°C and calcined at 450°C for 3 h. The precipitate was crushed and kept in a closed container [27].

### 2.3. Characterization of Synthesized CuO NP Material

The morphological features of CuO NPs were examined by field‐emission scanning electron microscopy (FESEM) (Shimadzu, Japan) and transmission electron microscopy (TEM) (Zeiss LEO 912 AB‐100 kv, Germany). The crystalline nature of CuO NPs was identified by X‐ray diffraction (XRD) by employing a Shimadzu 6000 diffractometer (Japan). Surface functional groups were identified by Fourier‐transform infrared (FTIR) spectroscopy (Shimadzu 8400, Japan) in the range of 4000–400 cm^−1^. The formation of CuO NPs was supported by spectral features with a UV‐Visible spectrophotometer, double beam, T80, England. A Brunauer–Emmett–Teller SSA method (TriStar II Plus 2.03) was employed to estimate specific SSA and pore parameters. The point of zero charge (pH_pzc_) of CuO NPs was established following a procedure reported by [28].

### 2.4. Experimental Procedure

The adsorption experiment of MV dye employed a 1000 mg/L MV stock solution that was subsequently diluted using deionized water to prepare the working solutions ranging from 20 to 140 mg/L. The pH of MV dye solution was adjusted to the desired values (pH between 3.58 and 10.53) using 0.1 M NaOH and HCl solutions.

A mixture was prepared using the dye solution of adjusted pH and synthesized CuO NPs powder (0.05 g/L) in a ratio of 10 mg/L of dye solution and 0.05 g/L CuO NPs powder in a 100‐mL conical flask. These flasks were corked and placed in a thermostatic water bath, stirred at a fixed rate of 150 rpm, and maintained at 298, 308, and 318 K until equilibrium was reached. After completion of each adsorption experiment, the mixtures were centrifuged (Hettich EBA‐20, Germany) at 3000 rpm for 5 min. The clear supernatant was measured on a T‐80 double‐beam UV‐VIS spectrophotometer (England) at 586‐nm wavelength. To account for the ideal operational conditions for the adsorption of MV dye, batch adsorption experiments were carried out by evaluating a number of parameters, such as CuO NPs dosage (0.02–0.25 g/L) and contact time (5–90 min).

The adsorption efficiency (AE%) and adsorption capacity (*q_eq_*, mg/g) of MV with CuO NPs powder were estimated by employing Equations (1) and (2), respectively:

(1) AE%=Co−CeqC0×100,

(2)  qeq=Co−Ceq×Vsolm.

*C*_0_ and *C*_*e* *q*_ are the initial and residual concentrations of MV (mg/L), *V*_*s* *o* *l*_ is the working volume (L), and *m* is the dosage of CuO NPs (g/L).

### 2.5. Reusability Investigation

Reusability is a significant parameter, which reflects an adsorbent′s ability for recovery over extended use in industrial processes. In turn, batch desorption experiments were conducted, where 0.05 g/L of CuO NPs was introduced into 50 mL of the original 60 mg/L MV solution and stirred continuously at 150 rpm and 298 K for 90 min over six desorption cycles. For each adsorption cycle to attain equilibrium, the surface of CuO NPs used in the previous cycle was washed with distilled water to remove any unbound MV dye, followed by use of 50 mL of 0.1 M HCl as the desorption agent. The residual unbound dye concentrations were measured after each cycle, and the desorption efficiency (DES%) was calculated by employing Equation (3):

(3) DES%=Desorbed dye mg/gAdsorbed dye mg/g×100.

## 3. Results and Discussion

### 3.1. Characterization of CuO NPs Adsorbent

#### 3.1.1. X‐ray Analysis

**Figure 1:**
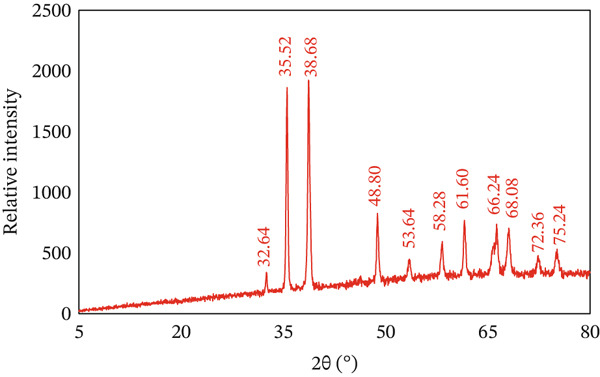
XRD pattern of the prepared CuO NPs adsorbent material.

XRD provides precise information on the crystalline structure and grain size of materials. CuO NPs were analyzed by X‐ray diffractometer using Cu K*α* radiation in the 2 theta 5°–80° range. The XRD peaks of CuO NPs appeared at variable 2 theta values (32.64°, 35.52°, 38.68°, 48.80°, 53.64°, 58.28°, 61.60°, 66.24°, 68.08°, 72.36°, and 75.24°) in agreement with (110), (−111, 111), (−202), (020, 202), (−113), (−311, 220), and (311, 004) planes, respectively, and are compatible with JCPDS Card No. 48‐1548 proving a single phase with a monoclinic framework as demonstrated in Figure 1 [29]. The presence of diffraction peaks (2 theta = 35°−38°) implied the formation of CuO NPs. CuO NPs are evidenced by XRD trends that offer evidence of CuO NPs crystalline sizes within the nanometer scale. Consequently, the current outcomes are similar to previous reports on CuO NPs synthesis [25].

#### 3.1.2. FESEM Analysis

**Figure 2:**
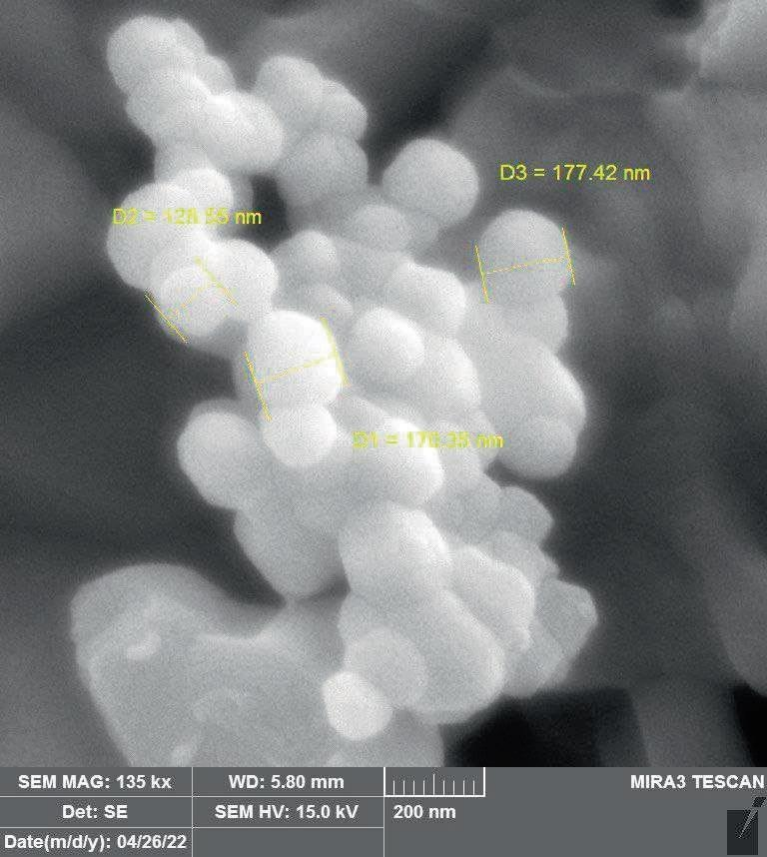
FESEM image of the CuO NPs adsorbent.

A FESEM map of the prepared CuO NPs was obtained (Figure 2), where the surface features of CuO NPs reveal a unique uneven geometry along with a high porosity. The presence of large cavities and open pores suggests that the CuO NPs adsorbent displays a high surface area, which enhances the adsorption properties and efficiency through an increased *q*_*e* *q*_ via greater interactions with MV [30]. The image clearly reveals that the prepared CuO NPs possess a semispherical morphology, in contrast to previous reports [31].

#### 3.1.3. TEM Analysis

**Figure 3:**
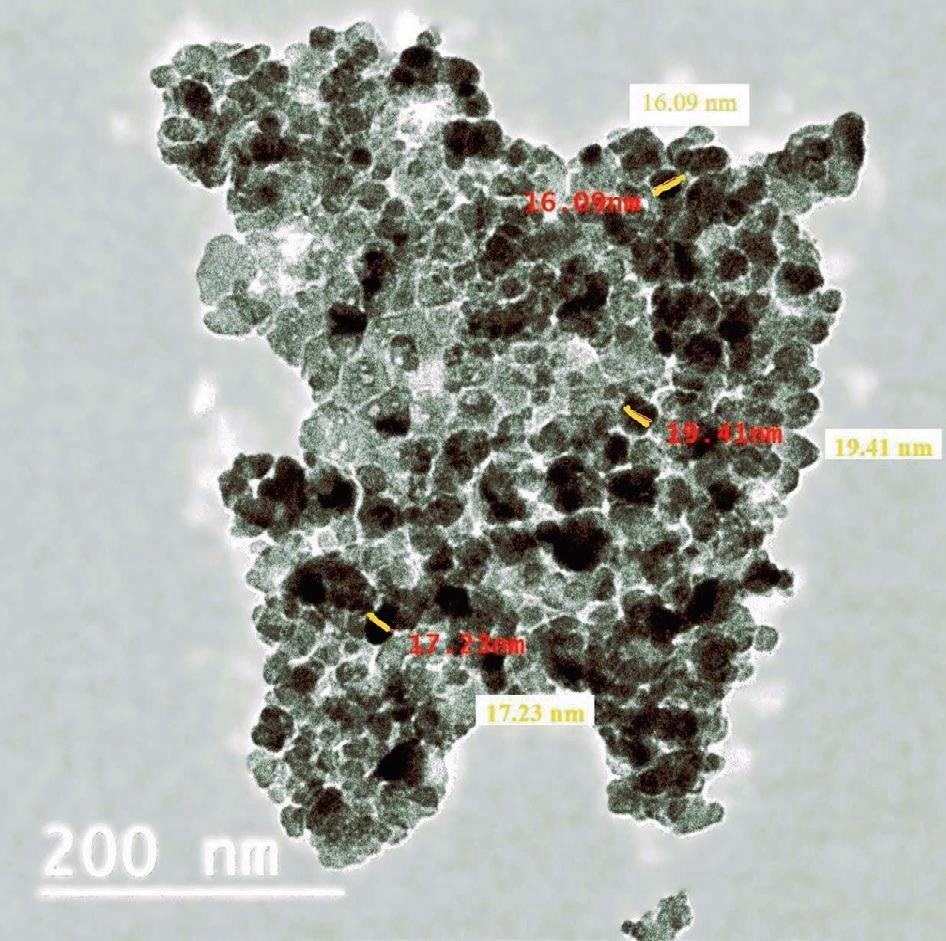
TEM images of the CuO NPs system.

Figure 3 illustrates the TEM micrograph of CuO NPs with primarily spherical particles. The well‐defined structure edges examined through TEM demonstrated the crystalline characteristic of CuO NPs. Figure 3 confirms the results of the FESEM analysis [32].

#### 3.1.4. FTIR Analysis

**Figure 4:**
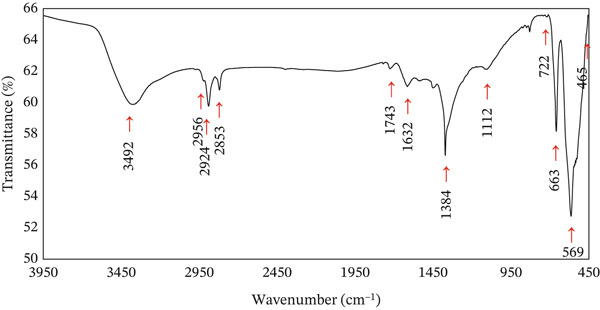
FTIR spectra of CuO NPs with the MV adsorption process.

The FTIR analysis for CuO NPs with MV dye (Figure 4), a specific absorption broad band (–OH– and –NH– vibrations), was noted at 3429 cm^−1^. Several bands occur at 2956, 2924, and 2853 cm^−1^, corresponding to C–H stretching vibrations. The IR band at 1632 cm^−1^ is due to C=C stretching of the aromatic ring structures of the dye and primary amide groups. An IR band at 1384 cm^−1^ is due to the C–O group stretching, whereas the band at 1112 cm^−1^ is due to the stretching vibration of the C–O–H bond. A distinct peak at 1743 cm^−1^ is characteristic of the C=O stretching vibration of the –COOH groups [33, 34]. In addition, the bands at 722, 663, 569, and 465 cm^−1^ are attributed to Cu–O vibrational modes [4]. This could be clarified by electrostatic interactions between MV cations with active functional sites of the CuO NPs surface. Table 1 summarized the band positions and their assignments for MV removal by the CuO NPs.

**Table 1:** The band positions (cm^−1^) and their assignment for the MV adsorption onto CuO NPs.

Functional groups inferred from FTIR spectral data	Wave numbers (cm^−1^)
O–H	3429
N–H	
C–H	2956, 2924, and 2853
C=C	1632
C–O	1384
C–O–H	1112
C=O	1743
Cu–O	722, 663, 569, and 465

#### 3.1.5. BET Analysis

**Figure 5:**
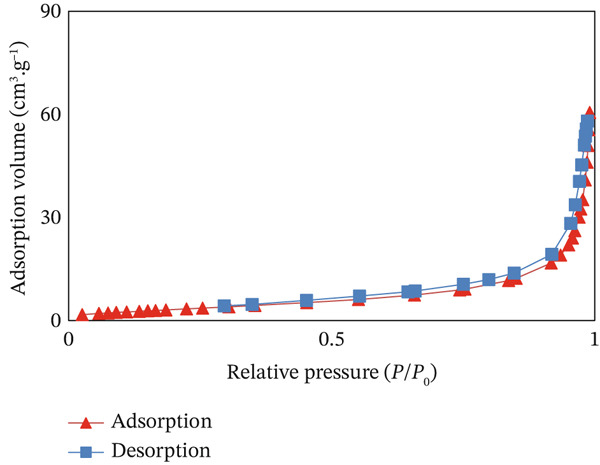
N_2_ adsorption–desorption isotherms of CuO NPs.

For the characterization of the CuO NPs surface properties, BET adsorption/desorption curve and pore structure characteristics are shown in Figure 5. Specific surface area (SSA) and average pore diameter were estimated from the nitrogen adsorption–desorption isotherm results at 77 K. The SSA of CuO NPs was estimated to be 12.97 m^2^·g^−1^, where its mean pore diameter was 28.55 nm, whereas the total pore volume is 0.0925 cm^3^·g^−1^. The adsorption–desorption isotherm is a Type IV system, according to the IUPAC system, which indicates that CuO NPs have mesoporous features [35, 36].

#### 3.1.6. UV‐VIS Spectrum

**Figure 6:**
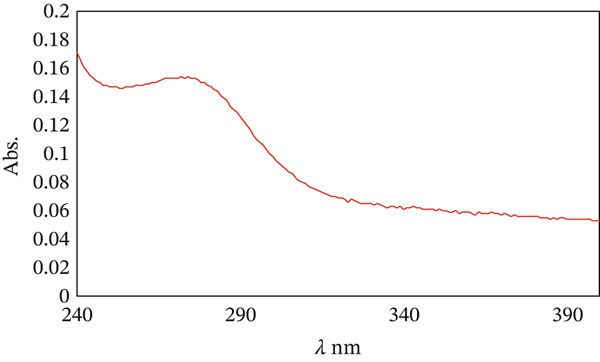
UV‐VIS spectrum of the prepared CuO NPs.

The UV‐VIS absorption spectrum of CuO NPs synthesized via *Beta vulgaris* L. extract is presented in Figure 6. For spectral analysis, 300 *μ*L of CuO NPs was mixed with 1 mL of distilled H_2_O. The broad peak at 275 nm confirms the formation CuO NPs, in agreement with the results reported in the literature [32].

### 3.2. Effect of CuO NPs Adsorbent Dosage on the Dye Adsorption

**Figure 7:**
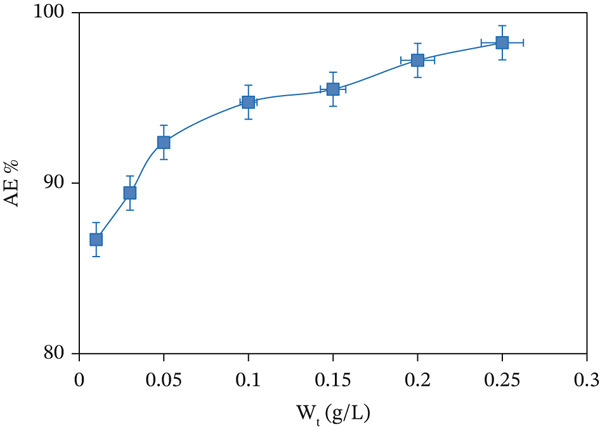
Effect of CuO NPs dosage on the AE (%) of MV dye at optimal conditions (298 K, initial pH = 4.54, MV concentration = 60 mg/L, and *t* = 90 min).

The CuO NPs dosage plays a crucial role in determining dye AE%. For the present study, the MV dye AE% was evaluated over a dosage range of 0.01–0.25 g/L for CuO NPs, an initial fixed concentration of 10 mg/L of dye, pH 4.54, 298 K, and a contact time of 90 min. As evidenced from Figure 7, AE% increased from 86.69% to 98.23% with increasing dosage (g/L) of CuO NPs. This occurs because the number of active sites is greater and there is more SSA for effective dye interactions. A 0.05 g/L dose of CuO NPs was selected to ensure saturation of the *q*_*e* *q*_ [37].

### 3.3. Effect of Contact Time

**Figure 8:**
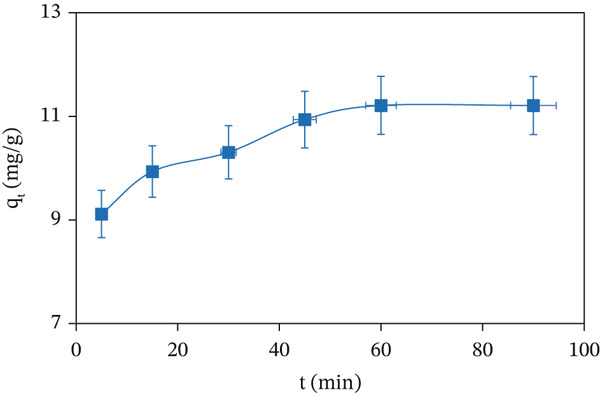
Effect of contact time on the adsorption capacity of MV dye at 298 K, initial pH = 4.54, and MV concentration = 60 mg/L.

The amount of MV adsorbed onto the CuO NPs surface was measured at variable contact times at 298 K, pH 4.54, and an initial dye concentration of 10 mg/L. In Figure 8, the *q*_*e* *q*_ of CuO NPs increased with longer contact times, attributed to greater numbers of MV dye molecules to interact and bind with the adsorbent surface. The rate of dye uptake was high initially, whereas equilibrium was reached ca. 90 min, but beyond the equilibrium point, there was no marked adsorption observed, indicating that the active sites on CuO NPs were saturated. Overall, the *q*_*e* *q*_ varied from 9.11 mg/g at 5 min to 11.20 mg/g at 90 min, which confirmed that 90 min was sufficient to complete the adsorption process [38].

### 3.4. Effect of pH Solution

**Figure 9:**
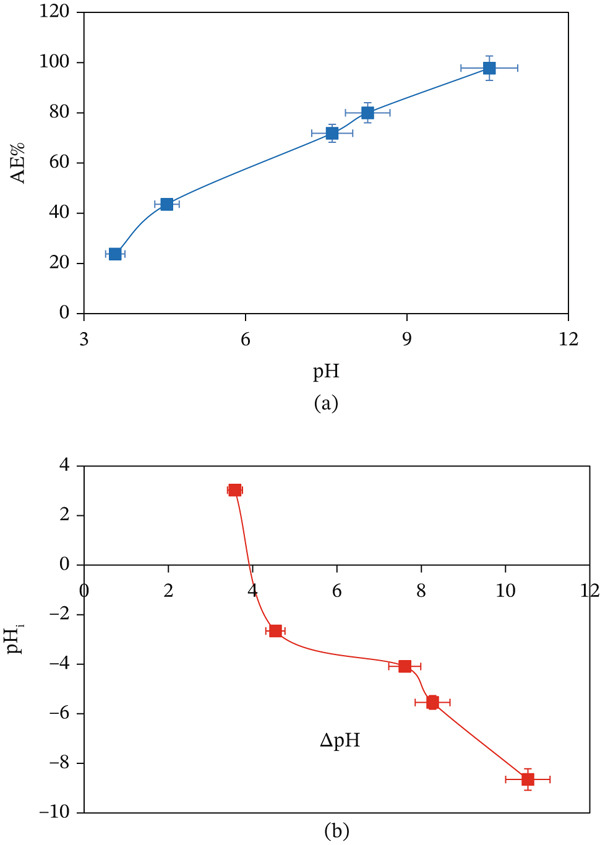
(a) Effect of pH on the AE (%) of the CuO NPs surface with the MV dye and (b) estimation of the pH_pzc_ of the CuO NPs surface at 25°C.

The pH of the solution is crucial for the adsorption process as it significantly influences the acidity or alkalinity of the medium, the adsorbent surface charge, and the chemical speciation of the liquid‐phase contaminants [39]. The effect of pH on the adsorption of MV onto CuO NPs was examined over a broad pH range (3.58–10.53), as shown in Figure 9a. The AE% was very low at acidic pH (23.74% at pH 3.58) but greatly increased with increasing pH, with the highest AE% of 97.76% at pH 10.53, revealing the high pH dependence on the adsorption process. The low AE% observed at acidic conditions is due to the high concentration of hydrogen ions (H^+^), which compete with the cationic MV dye molecules in filling active adsorption sites on the CuO NPs surface, resulting in an unfavorable adsorption process. Moreover, under acidic conditions, the adsorbent surface becomes protonated, leading to repulsive forces toward the positively charged MV ions, further preventing their adsorption.

Conversely, an elevated solution pH led to a greater AE% for CuO NPs, revealing a positive adsorptive condition. The observed enhancement relates to deprotonation of the CuO NPs surface, which enhances the electrostatic attraction between the negatively charged adsorbent surface and the MV cation dye. The adsorption behavior of MV is strongly linked with the pH_pzc_ for the CuO NPs adsorbent. In general, if pH < pH_pzc_, the surface of the adsorbent is positively charged. By comparison, the adsorbent surface is negatively charged at pH > pH_pzc_. In Figure 9b, the pH_pzc_ of CuO NPs is approximately 3.9, where the CuO NPs surface effectively adsorbs the cationic MV dye through electrostatic attraction since pH > pH_pzc_ [40]. Other studies have documented consistent outcomes related to pH and trends in the adsorbent surface charge [41, 42].

### 3.5. Kinetic Dye Adsorption Profiles

To investigate the dye adsorption kinetics, two types of models were studied: pseudo‐first‐order (PFO) and pseudo‐second‐order (PSO) models. The PFO model, proposed by Lagergren, is formulated by Equation (4) [43]:

(4) lnqeq−qt=Lnqeq−k1t.

The expression for the PSO model is defined by Equation (5) [44]:

(5) tqt=1k2qeq2+tqeq.

**Figure 10:**
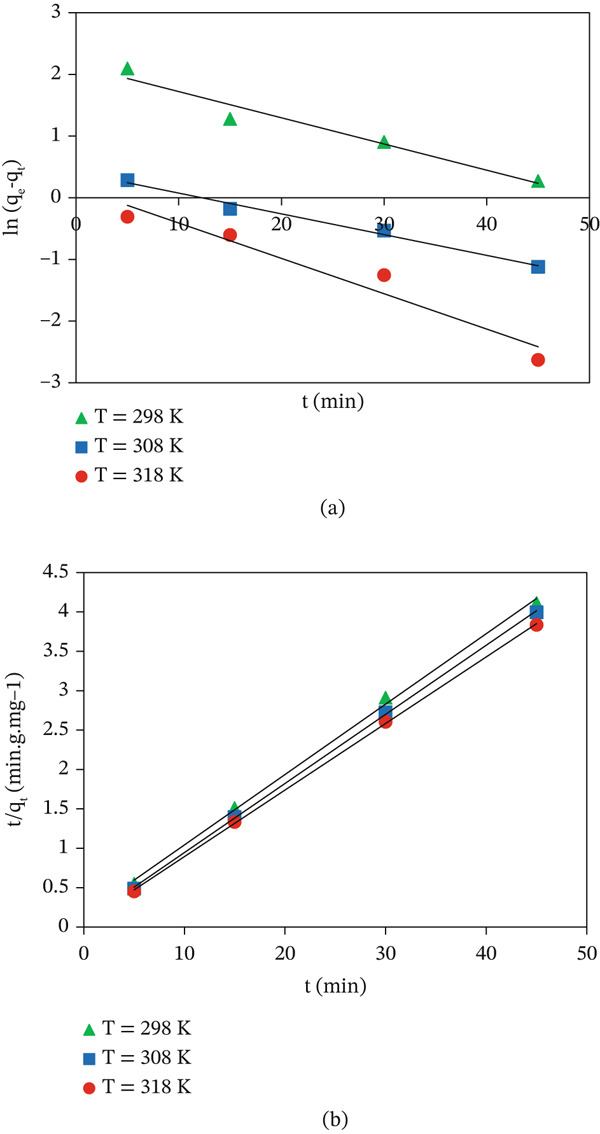
Linear regression curves of (a) PFO and (b) PSO kinetic curves for MV adsorption onto CuO NPs.

In this context, *q*_*t*_ and *q*_*e* *q*_ (mg·g^−1^) represent the *q*_*e* *q*_ of MV at a specific time (*t*) and at equilibrium, respectively, whereas *k*_1_ (min^−1^) and *k*_2_ (g·mg^−1^·min^−1^) are the rate constants for the PFO and PSO models. The respective curves of the two models are shown in Figure 10, and the kinetic parameters are listed in Table 2. The *R* ^2^ values in Table 2 clearly indicate that the PSO model describes the MV adsorption phenomenon onto CuO NPs more reliably. As well, the adsorption capacities calculated by the PSO model reveal close agreement with experimental values. This finding indicates that physisorption, resulting from attractions between the cationic groups of MV and the anionic groups of CuO NPs, provide the main mechanism governing MV adsorption [45].

**Table 2:** Kinetic adsorption parameters obtained from the PFO and PSO isotherm models.

Adsorbent	*T*(K)	*k*_1_(min^−1^)	*q*_*e*__,exp_ (mg/g)	*q*_*e*__,calc._ (mg/g)	*R* ^2^	*k*_2_(g mg^−1^ min^−1^)	*q*_*e*__,calc_ (mg/g)	*R* ^2^
CuO NPs	298	0.0425	11.21	5.83	0.9524	0.0539	11.19	0.998**3**
	308	0.0339	11.59	1.12	0.9871	0.1137	11.40	0.9998
	318	0.0316	11.81	0.44	0.9438	0.1469	11.83	0.9998

### 3.6. Adsorption Isotherm Analysis

The equilibrium adsorption data of MV on CuO NPs at variable temperatures were modeled using three isotherm models: Langmuir, Freundlich, and Temkin. These models were employed to provide the best fit results to the experimental data and to verify if the adsorption process occurs onto a homogeneous or heterogeneous surface. The Langmuir model (Equation 6) assumes that the process adopts a monolayer surface profile, where the adsorption sites are homogeneous and have similar energy [46].

(6) Ceqqeq=1qmaxKL+Ceqqeq

*K*_*L*_ (L/mg) is the Langmuir equilibrium constant. *q*_max_ and *K*_*L*_ can be estimated from the slope (1/*q*_max_) and intercept (1/*q*_max_ *K*_*L*_) for the linear plot of *C*_*e* *q*_/*q*_*e* *q*_ and *C*_*e* *q*_.

The Freundlich model (Equation 7) assumes that the adsorbent surface is heterogeneous and adsorption may also occur in multilayer formation [47].

(7) logqeq=logKf+1nlogCeq

Here, the Freundlich constants (*K*_*f*_, mg/g) and adsorption intensities (1/*n*) were calculated from the magnitudes of slope and intercept in the plot of logarithmic *q*_*e* *q*_ against the logarithmic equilibrium dye concentration (*C*_*e* *q*_) for MV.

The Temkin isotherm (Equation 8) assumes the following: (i) the heat of adsorption for all the molecules in the layer decreases linearly with coverage, due to interactions between the adsorbent surface and the bound adsorbate, and (ii) the adsorption process is distinguished by a uniform distribution of binding energies up to a maximal binding energy [48].

(8) qeq=BlnAT+BlnCeq

The parameter *B* = (*R* *T*/*b*), where *b* implies the Temkin constant and corresponds to the heat of adsorption (J/mol), *A* is the Temkin isotherm constant (L/g), *R* is the gas constant (8.314 [J/mol K]), and *T* is the temperature (K), whereas the values of *B* and *A*_*T*_ can be estimated from the slope and intercept in a plot of *q*_*e* *q*_ via ln *C*_*e* *q*_.

Based on the *R* ^2^ values in Table 3, the Freundlich isotherm model revealed a best fit to the adsorption data at equilibrium. The Freundlich model provided a better correlation with the experimental data than the Langmuir and Temkin models, with the *R* ^2^ values (0.98–0.99). There is strong agreement indicating that the surface of CuO NPs has heterogeneous active sites, which is described by the Freundlich isotherm for the MV dye adsorption by the CuO NPs adsorbent system [49]. The magnitudes of (1/*n*) in Freundlich profile were less than 1 indicating that the adsorption of MV by CuO NPs is a physical process and favorable overall. Additionally, the values of *K*_*F*_ rise when heightening the temperature, which might prove that the adsorption profile is endothermic [50].

**Table 3:** Model parameters of linearized Langmuir, Freundlich, and Temkin isotherms for MV adsorption by CuO NPs.

Adsorbent	Langmuir			
CuO NPs	*T* (K)	*K*_*L*_ (L/mg)	*q*_max_ (mg/g)	*R* ^2^
	298	0.0048	285.70	0.8542
	308	0.0091	383.10	0.6013
	318	0.0346	153.80	0.2202
	Freundlich			
	*T* (K)	*K*_*F*_ (mg/g)	1/*n*	*R* ^2^
	298	2.88	0.96	0.9997
	308	3.55	0.94	0.9990
	318	5.06	0.98	0.9881
	Temkin			
	*T* (K)	*B* (J/mol)	AT (L/g)	*R* ^2^
	298	0.390	2.17	0.9985
	308	0.085	2.23	0.9164
	318	0.083	10.10	0.9719

Comparison of the CuO NPs adsorption properties with other materials reported in the literature for MV removal from aqueous media reveals that CuO NPs possess a very high maximum *q*_*e* *q*_ relative to many other adsorbents, as noted in Table 4. The CuO NPs display superior adsorption toward MV, as compared with many other adsorbents, revealing its utility as an efficient and an alternative adsorbent for the removal of MV dyes from water.

**Table 4:** Comparison of MV dye adsorption capacities for MV with other adsorbents in aqueous media.

Adsorbent	*T*(K)	*q*_max_(mg/g)	pH	Kinetic model	Isotherm model	Adsorbent dosage (mg/L)	Reference
AgI/TiO_2_	303	4.12	**—**	Pseudo‐first order (PFO)	Freundlich	0.01	[51]
TiO_2_	303	3.00	**—**	Pseudo‐first order (PSO)	Langmuir	0.01	[51]
Sepiolite	303	0.18–0.26	7	**—**	Langmuir	0.25	[52]
Banana peel	303	1.08	6–7	Intraparticle diffusion	Freundlich	0.001	[53]
CuO NPs	318	5.06	4.54	Pseudo‐second order (PSO)	Freundlich	0.05	Current work

### 3.7. Adsorption Thermodynamics and Activation Energy

Thermodynamic factors, including standard Gibbs free energy change (*Δ*G°), standard enthalpy change (*Δ*H°), and standard entropy change (*Δ*S°), were determined by employing Equations (9)–(11) [54]:

(9) ΔGo=−RTLnKd,

(10) Kd=Co−CeqCo×vsolW,

(11) LnKd=−ΔHoRT+ΔSoR.

**Figure 11:**
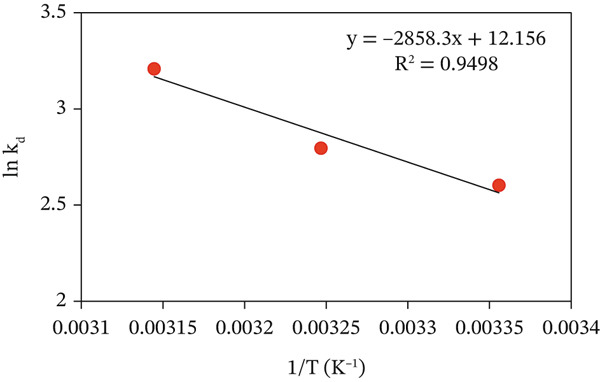
van′t Hoff plot for adsorption of MV dye onto CuO NPs adsorbent.

The estimated thermodynamic parameters (Table 4) are listed for the adsorption of MV onto CuO NPs. The values were derived from a plot of ln *K*_*d*_ against 1/*T* (K^−1^), as shown in Figure 11, where the slope and intercept were proportional to *Δ*H° and *Δ*S°, respectively. The influence of temperature (298, 308, and 318 K) on CuO NPs‐MV dye adsorption interaction was investigated with fixed values of other parameters (pH, adsorbent dosage, and dye concentration). Generally, the results indicate that the *q*_*e* *q*_ of CuO NPs increased with temperature, which means that the process is more favorable at elevated temperatures. This increase is more likely due to reduced solution viscosity and higher kinetic energy of MV dye species, which allow them to diffuse within the pores of the CuO NPs [33].

Negative *Δ*G° values occur at all temperatures and confirm the spontaneous nature of adsorption of MV onto CuO NPs. Also, the negative trend of *Δ*G° with increasing temperature further confirms that higher temperature facilitates a greater level of adsorption. The positive *Δ*H° signifies that the process is endothermic in nature, and the positive value for *Δ*S° signifies an entropy‐driven process due to greater randomness at the solid–liquid interface upon dye adsorption as noted in Table 5 [55]. The Arrhenius Equation (12) calculated the activation energy (*E*_*a*_) of the adsorption process.

(12) Lnk2=LnA−EaRT 

**Table 5:** Thermodynamic parameters estimated for MV adsorption onto the CuO NPs adsorbent.

Adsorbent	*T* (K)	Ln *K*_*d*_	ΔG° (kJ/mol)	ΔH° (kJ/mol)	ΔS° (J/mol·K)
CuO NPs	298	2.60	−6.5	23.8	101.1
	308	2.79	−7.2		
	318	3.21	−8.5		

**Figure 12:**
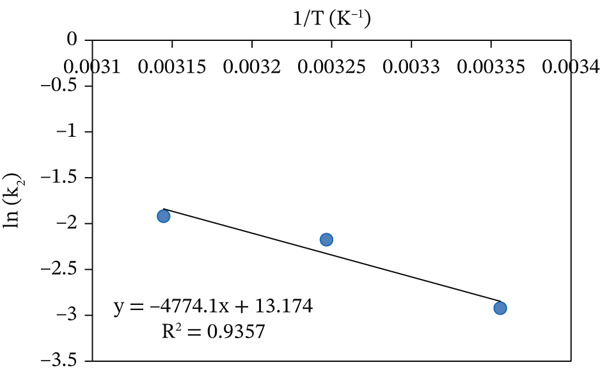
Plot of ln *k*_2_ versus 1/*T* for estimation of activation energy at variable temperature.

The value of *E*_*a*_ for the adsorption of MV onto the CuO NPs was determined from the slope of the linear plot of ln *k*_2_ versus 1/*T*, presented in Figure 12. The activation energy was determined to be 39.7 kJ/mol, which indicates that the adsorption process takes place primarily through a physisorption mechanism [36].

### 3.8. Repeatability Performance

**Figure 13:**
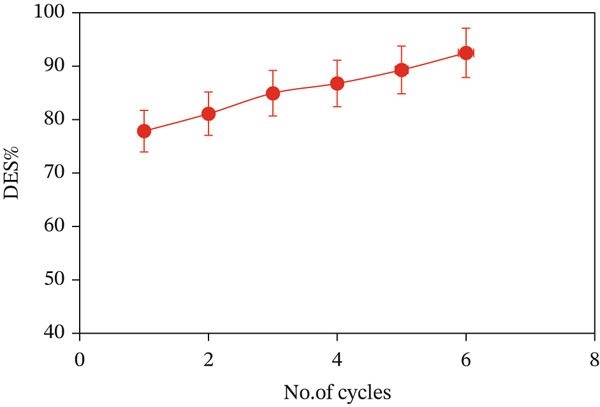
Batch desorption cycles after regeneration with 0.1 M HCl (experiment conditions: 298 K, 150 rpm, *C*_0_, MV = 60 mg/L, and pH 4.54).

From an economic perspective, the recyclability of an adsorbent is arguably one of the most significant parameters in ascertaining its effectiveness in practical applications. It represents the recoverability of a used (spent) adsorbent and reusability within multiple cycles of adsorption/desorption upon proper treatment. Acid treatment used in this work was found to be an effective method to desorb MV dye from CuO NPs saturated surface. As Figure 13 clearly shows, CuO NPs maintained a high desorption efficiency (DES%) of approximately 92.49% after six consecutive cycles. The results suggest that the adsorption interaction between MV dye species and CuO NPs is governed predominantly by weak interactions. Moreover, the increased accumulation of dye molecules in the higher cycles may have weakened their interaction with the adsorbent, making desorption more efficient. These findings warrant the use of CuO NPs as an economic and recyclable adsorbent for the removal of cationic or basic dyes from aqueous media [56, 57].

### 3.9. Adsorption Mechanisms of MV Dye Onto the CuO NPs Adsorbent

Mechanistically, the presence of a range of adsorptive groups on the surface of the adsorbent is significant in controlling the adsorption process. Of special importance is a higher ratio of active heteroatom‐containing groups, which enhances the interaction between the adsorbent and the adsorbate contaminant. Generally, it is observed that adsorption tends to be a multimodal process, which includes physisorption, ion exchange, and coordination, among other contributions. Such mechanisms are governed by the functional groups of the adsorbent, the nature of contaminants, and the ionic strength of the media. Various reactive functional groups present on the CuO NPs surface are inferred to contribute cooperatively to MV dye adsorption via multifunctional interactions. The proposed mechanisms for the adsorption of MV dye onto CuO NPs involve several types of interactions: (1) electrostatic attraction, (2) hydrogen bonding, (3) ion exchange, (4) Lewis′s acid–base interactions, and (5) *π*–*π* interactions. Since MV is a cationic (positively charged) dye, it is electrostatically attracted to the negatively charged functional groups of CuO NPs (e.g., –COOH and –OH) at pH higher than the pH_PZC_ (3.9). Further, hydrogen bonds between hydrogen‐donor groups like –COOH on CuO NPs and hydrogen‐acceptor sites in the aromatic ring structure of MV can be formed.

**Figure 14:**
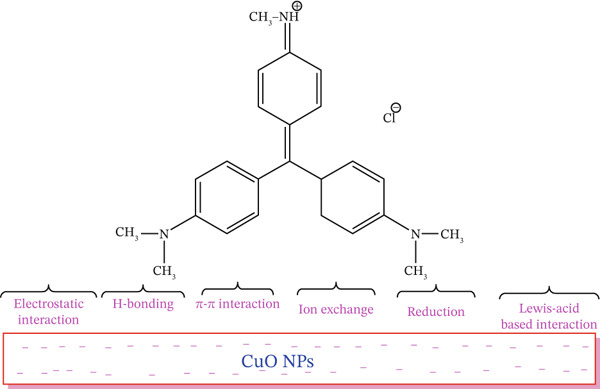
Proposed contributions to the adsorption process for MV dye onto the CuO NPs sorbent.

Ion exchange is also involved in the removal process, where it is catalyzed by hydroxyl (–OH) and carbonyl (C=O) groups on the CuO NPs surface [58]. Besides, the N‐atoms of the MV dye serve as Lewis base sites, which can bind with Cu^2+^ ions on the CuO NPs surface, forming Lewis′s acid–base interactions [59]. The synthesis atmosphere of CuO NPs (i.e., nitrogen or oxygen atmosphere) also influences its activity. Such synergistic interactions are potential contributions to the overall adsorption mechanism, as illustrated in Figure 14.

## 4. Conclusion

In this study, CuO NPs were synthesized along with *Beta vulgaris* L. extract and employed as an effective adsorbent for the elimination of the basic dye MV from aqueous media. Different characterization techniques such as FTIR, TEM, XRD, FESEM, and BET surface area were employed that confirmed the porous and crystalline structure of the CuO NPs adsorbent. The abundant negatively charged functional groups of CuO NPs surface conducted a predominant role in promoting the MV depollution via hydrogen bonds and electrostatic interactions at basic system (pH > pH_pzc_). The FESEM image displayed the existence of a noticeable porosity and great holes with individual morphology. The predicted crystalline structure from the FESEM and TEM findings agreed well. The attained XRD patterns resulted the existence of obvious crystallographic phases of the synthesized CuO NPs. UV‐VIS spectral results for CuO NPs affirmed the synthesis with an absorbance band at 275 nm. The CuO NPs possessed a surface area of 12.97 m^2^/g with dye *q*_*e* *q*_ of 5.06 mg/g under the following optimal conditions: initial pH 4.54 (natural), 318 K, contact time of 90 min, initial dye concentration of 60 mg/L, and CuO NPs dosage of 0.05 g/L. Among the isotherm models investigated, the Freundlich model provided the best fit results to the experimental data. This trend provides evidence of multilayer adsorption onto a heterogeneous surface. The PSO model favorably described the kinetics of dye adsorption. The negative values of *Δ*G° for the dye adsorption confirmed the spontaneous nature of the process, whereas positive values for enthalpy (*Δ*H°) and entropy (*Δ*S°) signified that the adsorption was endothermic and entropy driven, in accordance with greater molecular disorder at the solid–liquid interface. Remarkably, CuO NPs retained 92.49% of the AE% up to six cycles of reuse. This is consistent with the proposed adsorption mechanisms, and the activation energy indicates that the system is physisorption. The application of CuO NPs as an adsorbent in the treatment of wastewater samples demonstrated its high potential for practical dye removal in water treatment processes. This study demonstrates that CuO NPs produced from *Beta vulgaris* L. extract using a green precipitation method are an environmentally friendly, low‐cost, and recyclable adsorbent with high potential to eliminate dyes in water. These results enforce the potential of them as a practicable aid material for treating industrial wastewater.

## Author Contributions

Dhafir T.A. AL‐Heetimi, Muna Abd Ul Rasool AL‐Kazragi, and Amal Khudair Al‐Jaafari designed the experiments and performed the analytical work. Together with Lee D. Wilson, they contributed to writing the original draft of the manuscript.

## Funding

No funding was received for this manuscript.

## Disclosure

All authors reviewed and approved the final version of the manuscript.

## Conflicts of Interest

The authors declare no conflicts of interest.

## Data Availability

The data that support the findings of this study are available from the corresponding author upon reasonable request.
